# Supplementary material for: Building block aspect ratio controls assembly, architecture, and mechanics of synthetic and natural protein networks
Source: Nat Commun. 2023 Sep 11;14:5593. doi: 10.1038/s41467-023-40921-7 (PMC10495373; doi:10.1038/s41467-023-40921-7)
Supplement: Supplementary file 1 — Supplementary Information [file 41467_2023_40921_MOESM1_ESM.pdf]

Supplementary Information

Building Block Aspect Ratio Controls Assembly, Architecture, and Mechanics of Synthetic and Natural Protein Networks

Matt D G Hughes[1], Sophie Cussons[2,3], Benjamin S Hanson[1], Kalila R Cook[1], Tímea Feller[4], Najet Mahmoudi[5], Daniel L Baker[1], Robert Ariëns[4], David A Head[6], David J Brockwell[2,3], Lorna Dougan*[1,2]

1 School of Physics and Astronomy, Faculty of Engineering and Physical Sciences, University of Leeds, UK
2 Astbury Centre for Structural Molecular Biology, University of Leeds, UK
3 School of Molecular and Cellular Biology, Faculty of Biological Sciences, University of Leeds, UK
4 School of Medicine, Faulty of Medicine, University of Leeds, UK
5 ISIS Neutron and Muon Spallation Source, STFC Rutherford Appleton Laboratory, Oxfordshire, UK
6 School of Computing, Faculty of Engineering and Physical Science, University of Leeds, UK
Corresponding Author: Lorna Dougan, L.Dougan@leeds.ac.uk

Derivation of Rotating Rod Model (Eqn. 1), also known as the transition from the dilute to semi-dilute regime for rod-like particles

Here we consider a suspension of N cylindrical particles of length, L, and diameter, d in a volume, V, such that the volume fraction, ϕ, equals

$$\phi = \frac{N \cdot v_p}{V} = \frac{v_p}{v_a}$$

Where v_p is the volume of the cylindrical particular and v_a is the available volume per particle. We can say that a cylindrical particle is able to freely rotate about its centre of mass if

$$L < \sqrt[3]{v_a}$$

i.e. the length of the rod is shorter than the length of the cubic voxel of available volume. Substituting $v_a = \frac{v_p}{\phi}$ and $v_p = \frac{\pi}{4} d^2 L$ yields

$$L < \sqrt[3]{\frac{\pi}{4\phi} d^2 L}$$

Here we define the aspect ratio as AR = L/d, substitute L = AR·d and rearrange

$$AR^2 < \frac{\pi}{4\phi}$$

Equating both sides would yield the critical point beyond which the cylindrical particle is no longer able to freely rotate without colliding with another cylindrical particle.

$$AR_{crit}^{rod} = \sqrt{\frac{\pi}{4\phi}}$$

Determination of Fibrin Protofibril Aspect Ratio

The critical length of fibrin protofibrils is between approximately 20-25 monomer units of fibrin[1]. Considering 45 nm monomer length and the double-stranded structure, protofibril length for 20-25 monomers is 450-563 nm while protofibril width is 10-13 nm, taking into account the 8 nm distance between the parallel molecular axes of protofibrils[2] and the 2-5 nm monomer width of fibrin monomers[3]. These ranges give an average length and width of fibrin protofibrils of 506.5 nm and 11.5 nm, respectively, which would correspond to an average aspect ratio for fibrin protofibrils of 44.

Supplementary Tables

Protein L construct	Scattering Length Density in 100% D_2O (x10^{-6} A^{-2})
pL_1	3.227
pL_2	3.211
pL_3	3.194
pL_4	3.185
pL_5	3.18
pL_6	3.176
pL_7	3.173

Supplementary Table 1: Protein L constructs and their associated scattering length densities in 100% D_2O polyprotein SLD values. Note SLD of D_2O = 6.376 x10^{-6} A^{-2}.

**Parameter**	**25mg/ml**	**37.5mg/ml**	**50mg/ml**
α	0.5 ± 0.1	0.571 ± 0.003	0.7 ± 0.1
k_z	3.5 ± 0.5	7.11 ± 0.02	7 ± 0.7
AR_z	3.1 ± 0.2	2.8 ± 0.01	2.9 ± 0.5
k_c	1.1 ± 0.2	2.747 ± 0.007	3 ± 0.8
AR_c	6.2 ± 0.7	5.13 ± 0.01	4 ± 0.3
G'(∞)	10 ± 2	20.75 ± 0.01	36 ± 1
Reduced Chi squared	0.02699	4.91111E-8	3.11684

Supplementary Table 2: Fitted parameters with associated fit error from equation 4, extracted from the storage modulus vs AR data in figure 1b. Additionally the reduced chi-squared values of these fits are listed.

25 mg·ml^{-1}			37.5 mg·ml^{-1}			50 mg·ml^{-1}		
pL Aspect Ratio	Protein Volume Fraction (%)	Water Volume Fraction (%)	pL Aspect Ratio	Protein Volume Fraction (%)	Water Volume Fraction (%)	pL Aspect Ratio	Protein Volume Fraction (%)	Water Volume Fraction (%)
1	1.82	98.18	1	2.74	97.26	1	3.65	96.35
2	1.82	98.18	2	2.74	97.26	2	3.65	96.35
3	1.82	98.18	3	2.74	97.26	3	3.65	96.35
4	1.82	98.18	4	2.74	97.26	4	3.65	96.35
5	1.82	98.18	5	2.74	97.26	5	3.65	96.35
6	1.82	98.18	6	2.74	97.26	6	3.65	96.35
7	1.82	98.18	7	2.74	97.26	7	3.65	96.35

Supplementary Table 3: The protein and water volume fraction of protein L construct hydrogels as a function of pL construct aspect ratio and at varying protein concentrations in mg·ml^{-1}, equivalent to those show in figure 1b,d and 2d.

Protein L 7-Mer			Fibrinogen		
Protein Conc (mg·ml^{-1})	Protein Volume Fraction (%)	Water Volume Fraction (%)	Protein Conc (mg·ml^{-1})	Protein Volume Fraction (%)	Water Volume Fraction (%)
6.25	0.46	99.54	0.1	0.01	99.99
12.5	0.91	99.09	0.23	0.02	99.98
25	1.82	98.18	0.5	0.04	99.96
37.5	2.74	97.26	0.75	0.05	99.95
50	3.65	96.35	1	0.07	99.93
			1.5	0.11	99.89
			2.2	0.16	99.84

Supplementary Table 4: The concentrations in mg·ml^{-1} of Protein L 7-mer and Fibrinogen used in the lag time measurements (Fig. 3), with the corresponding protein and water volume fraction.

Supplementary Figures

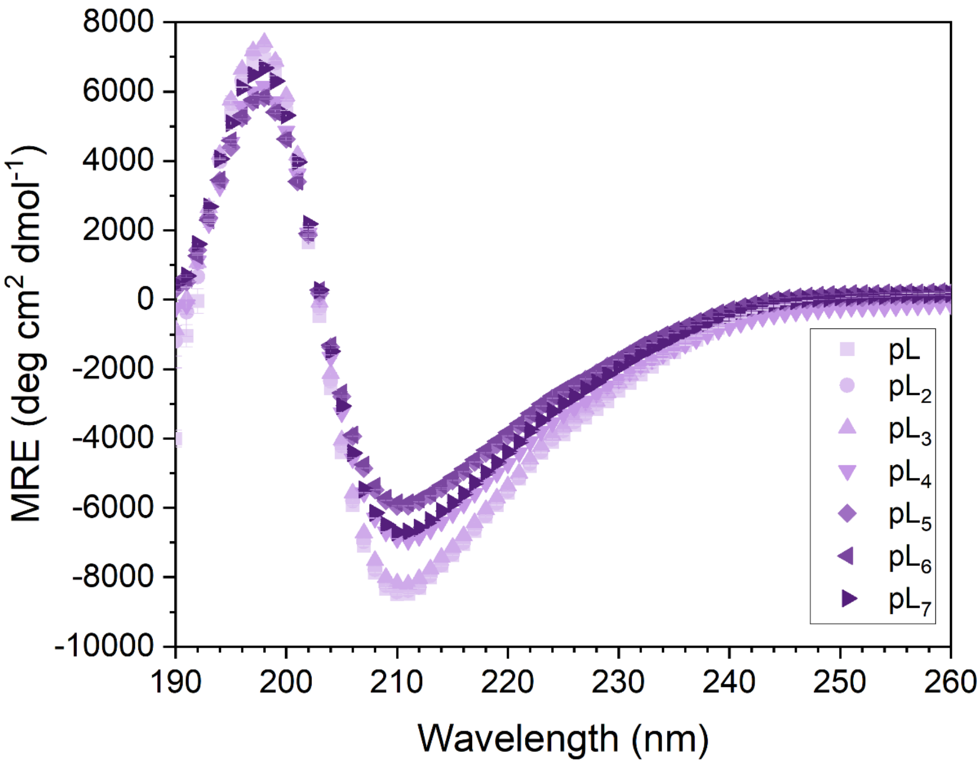

Supplementary Figure 1: Circular dichroism spectra (Mean Residue Ellipticity vs Wavelength) of pL and pL polyprotein constructs at approx. 0.2 mg/ml in 25mM phosphate buffer. All constructs exhibit a similar profile to the monomeric pL, demonstrating that the pL polyproteins are in the same folded state as the monomeric pL. Data points are presented as mean values +/- SEM, where n = 3.

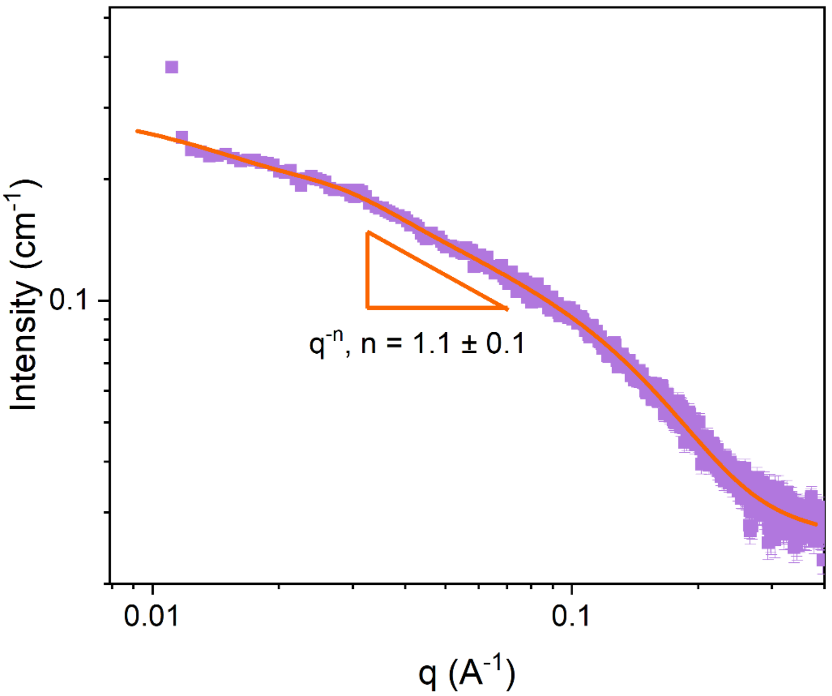

Supplementary Figure 2: SAXS curve (purple squares) of, intensity vs the scattering vector q, of pL_7 in 25mM phosphate buffer (pH 7.4) at a protein concentration of 10mg/ml at 20°C. The orange line shows the unified power fit (supplementary methods) to the SAXS data allowing for the extraction of the power law exponent in the mid-q region. Data points are the mean values of the linearly binned histograms, the y error bars show the standard error.

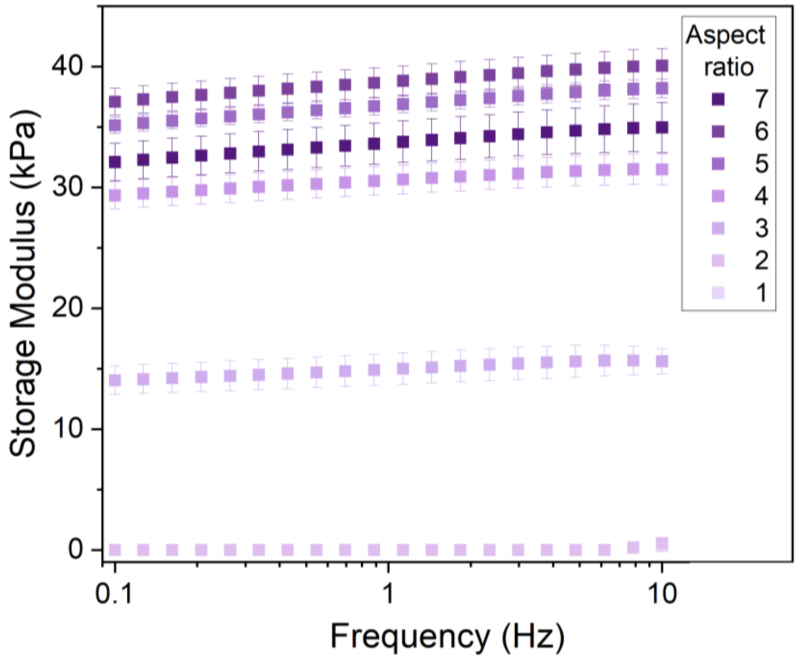

Supplementary Figure 3: Exemplar frequency sweeps showing the storage, G', of chemically cross-linked pL_x hydrogels (final concentrations: 50 mg/mL pL_x, 30 mM NaPS, 100 µM Ru(II)bpy_2^{3+}), where x is the number of domains in the building block and is synonymous with the aspect ratio of the building block. Data points are presented as mean values +/- SEM, where n = 3.

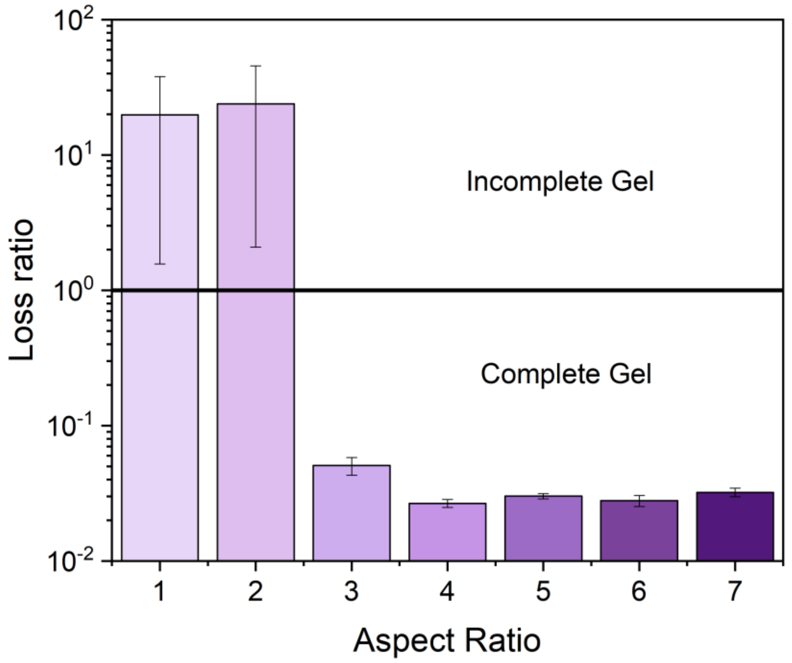

Supplementary Figure 4: Loss ratio of pL hydrogels as a function of building block aspect ratio (final concentrations: 50 mg/ml pL_x, 30 mM NaPS, 100 µM Ru(II)bpy_3^{2+}). Columns display the calculated loss ratio, defined as G"/G', from the frequency sweeps (supplementary figure 3), where the error bar is the propagated standard error.

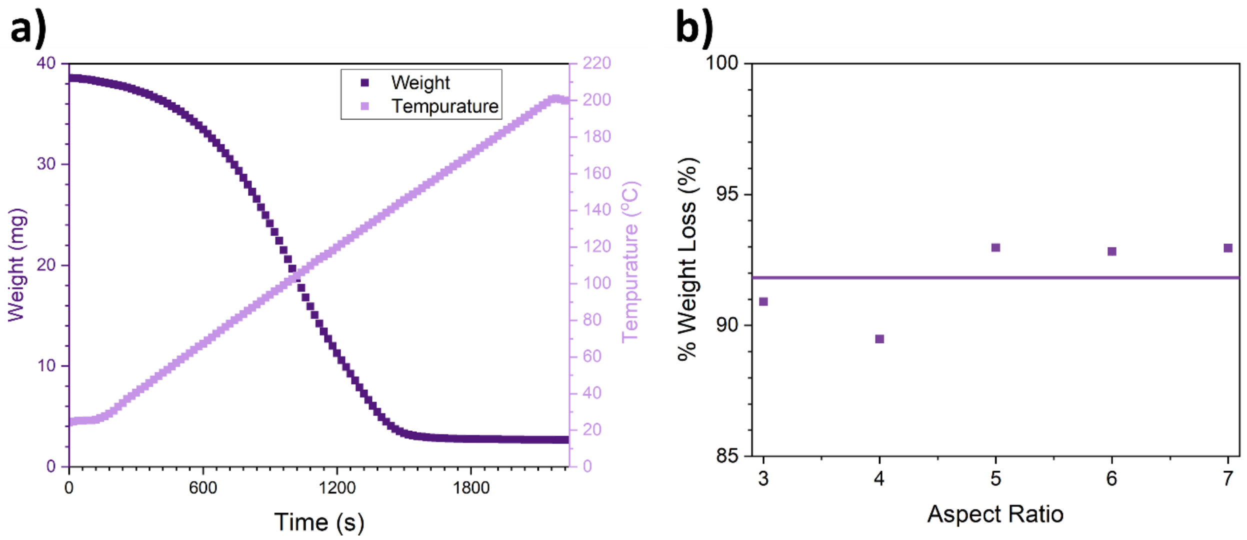

Supplementary Figure 5: **a)** Exemplar weight loss curves for pL hydrogels constructed from AR = 5 polyprotein constructs at 37.5mg/ml, where the weight (dark purple) is shown to decrease as the temperature (light purple) is increase, due to the water being evaporated. **b)** The extracted percentage weight loss (Supplementary Methods) as a function of building block aspect ratio (final concentrations: 50 mg/ml pL_x, 30 mM NaPS, 100 µM $Ru(II)bpy_3^{2+}$). The solid line shows the average value of all the points (91.8 ± 0.7%). Error bars denote the fitting error.

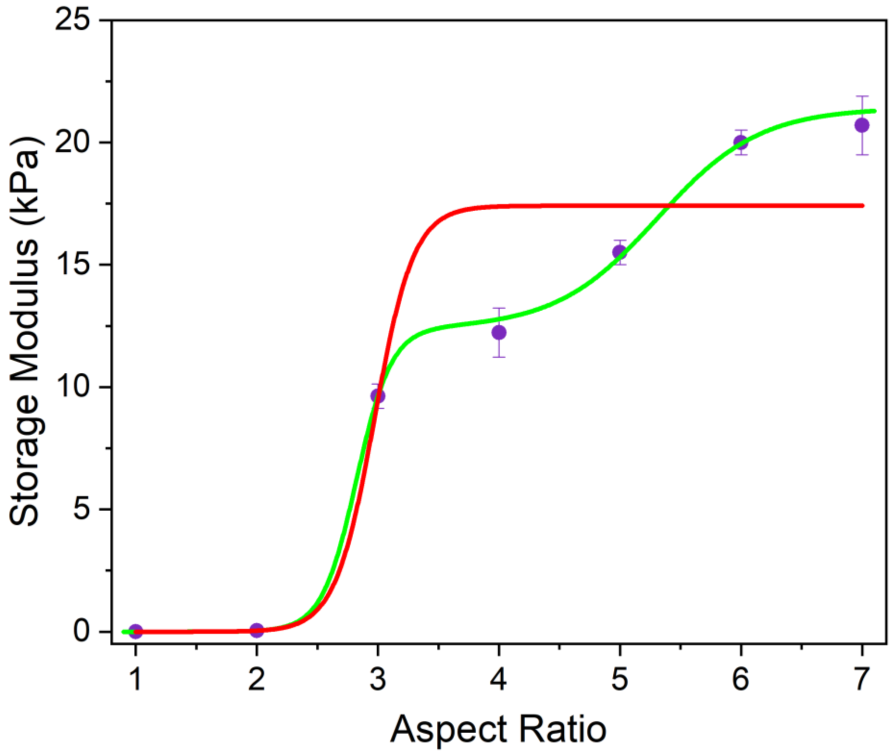

Supplementary Figure 6: Exemplar fittings of pL hydrogel storage moduli as a function of aspect ratio (final concentrations: 37.5 mg/ml pLx, 30 mM NaPS, 100 µM $Ru(II)bpy_3^{2+}$), using a single sigmoid function (red) and a dual sigmoid function (green). Data points are presented as mean values +/- SEM, where n = 3.

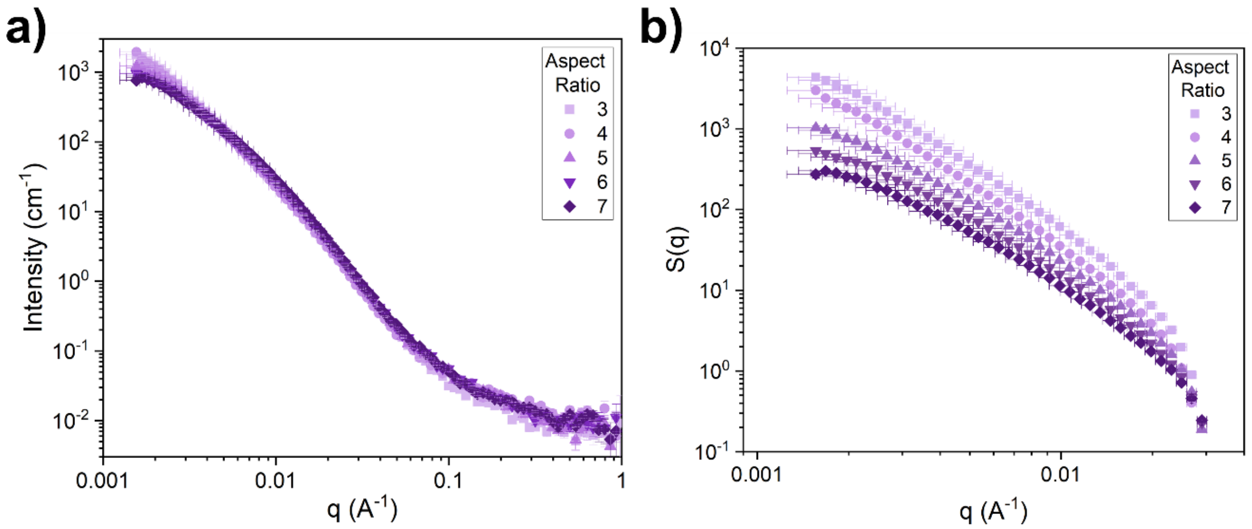

Supplementary Figure 7: a) SANS curves for pL hydrogels as a function of building block AR, at a protein concentration of 25mg/ml. Data points are the mean values of the logarithmically binned histograms, the y error bars show the standard error, while the x error bar is related to the detector resolution. **b)** Extracted structure factors for pL hydrogels as a function of building block AR, at a protein concentration of 25mg/ml. The error bars show the propagated errors from supplementary figure 6a.

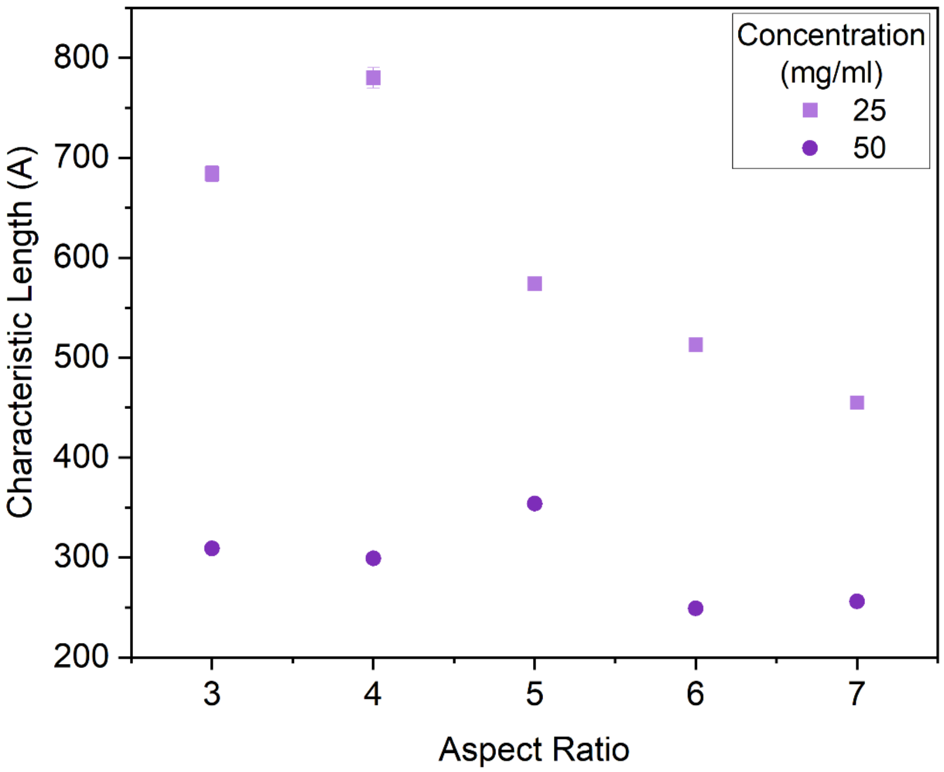

Supplementary Figure 8: The extracted correlation length of the fractal network of pL hydrogels at a protein concentration of 25mg/ml (light purple) and 50mg/ml (dark purple), as a function of building block aspect ratio. Error bars denote the fitting error.

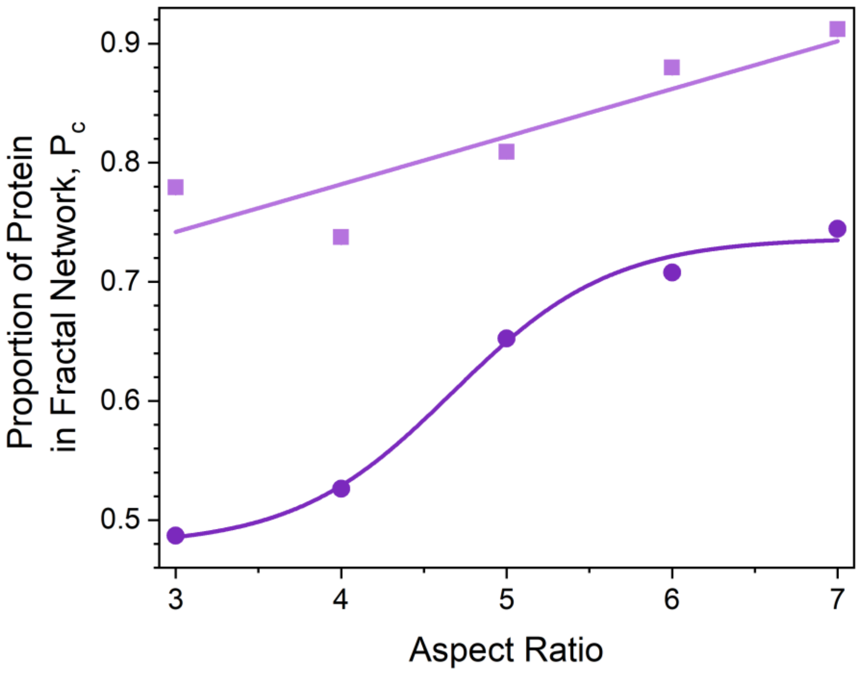

Supplementary Figure 9: The proportion of protein in the fractal network of pL hydrogels at a protein concentration of 25mg/ml (light purple) and 50mg/ml (dark purple), as a function of building block aspect ratio. Solid lines show a linear (light purple) and sigmoid (dark purple) fit to the 25 and 50 mg/mL data, respectively. Error bars denote the fitting error.

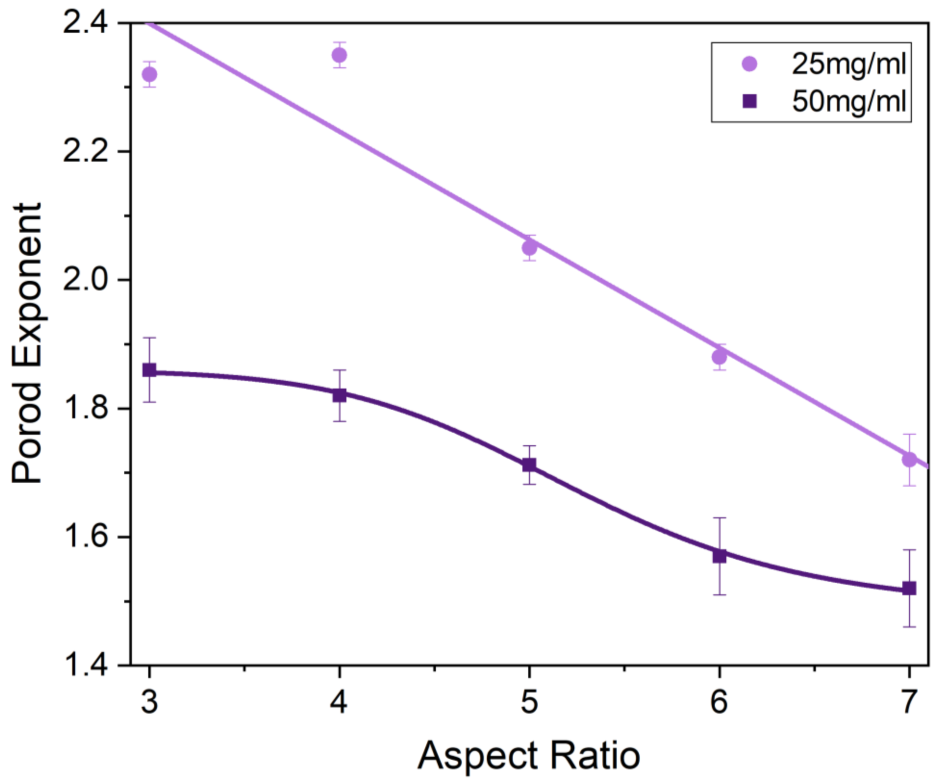

Supplementary Figure 10: The model independent Porod exponent extract from SANS data of pL hydrogels at a protein concentration of 25mg/ml (light purple) and 50mg/ml (dark purple), as a function of building block aspect ratio. Solid lines show a linear (light purple) and sigmoid (dark purple) fit to the 25 and 50 mg/mL data, respectively. Error bars denote the fitting error.

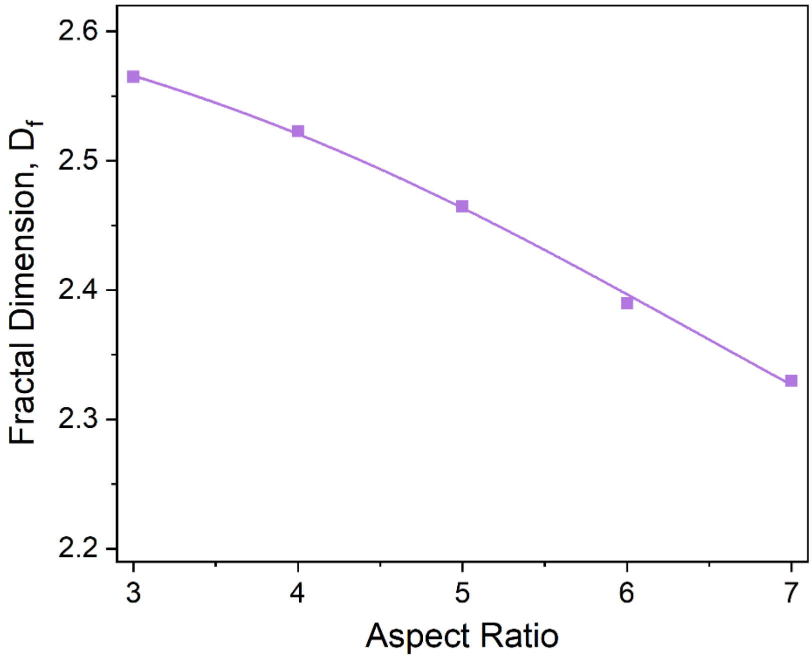

Supplementary Figure 11: Extracted fractal dimension of pL hydrogels as a function of building block AR at protein concentrations of 25mg/ml. The error bars here denote the fitting error of the fractal dimension to SANS curves. Solid line shows a sigmoid fit to the 25 mg/mL data. The $AR_{midpoint}$ of the fit is found to be 6.5 ± 0.3.

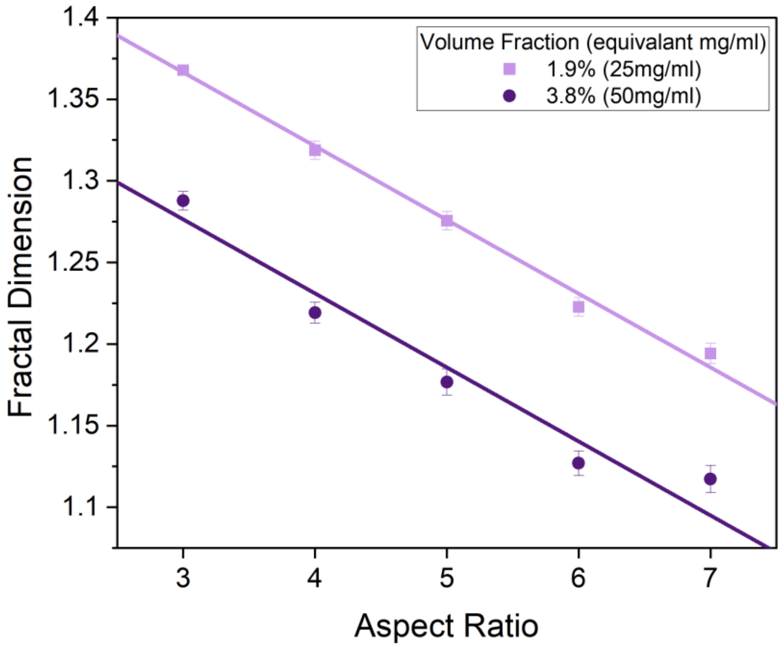

Supplementary Figure 12: Fractal dimension calculated by the box-counting episode of the kinetic lattice model simulation as a function of building block aspect ratio, at effectively volume fractions of 1.85% (light purple) and 3.7% (dark purple). Solid lines show linear fits to the 1.9% and 3.8% volume fraction data.
Note: The D_f values extract from the kinetic lattice model are approximately two-fold lower than the D_f values extracted from our SANS results (Fig. 2d). This systematic effect has previously been observed in other colloidal based simulations[4] and was attributed to 'on-lattice' simulations not accurately predicting the short-range (non-fractal regime). Data points are presented as mean values +/- SEM, where n = 10.

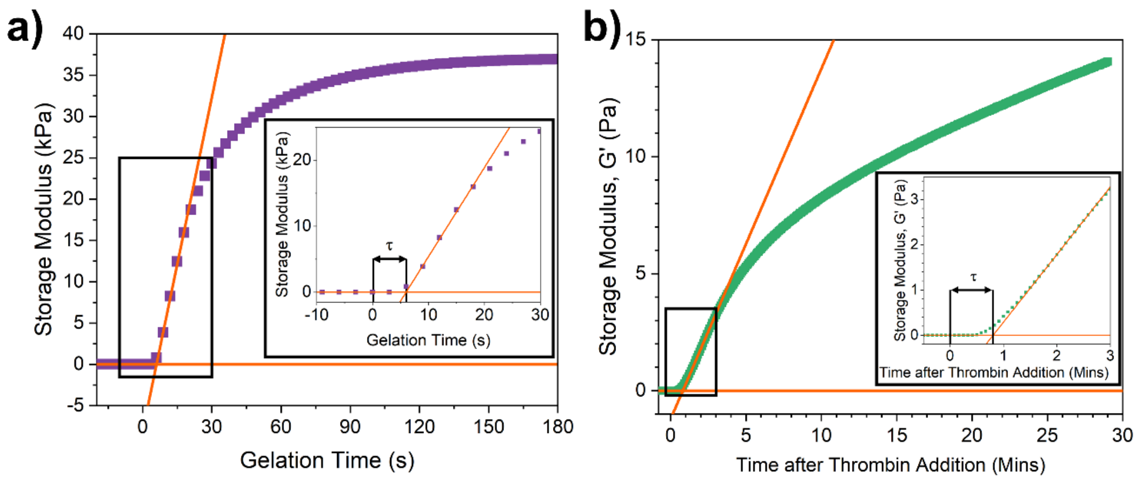

Supplementary Figure 13: a) Exemplar gelation curve of pL_7 hydrogels (at a protein concentration of 50mg/ml) showing the evolution of storage modulus over time. **b)** Exemplar gelation curve of fibrin networks (at a protein concentration of 2.2 mg/ml) showing the evolution of storage modulus over time after thrombin addition. The orange linear fit lines and insets in both panels demonstrate how the lag time, τ, are extracted from these curves.

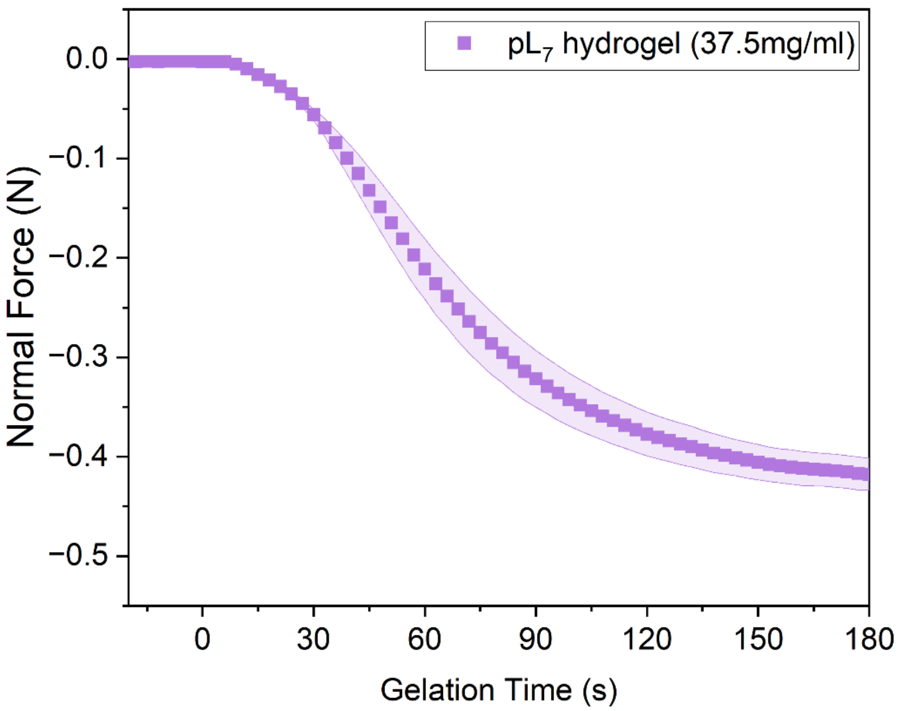

Supplementary Figure 14: Exemplar normal force of pL_7 hydrogel (at a protein concentration of 37.5mg/ml) during gelation via photo-chemical crosslinking. This curve is indicative of the pL hydrogels considered in this work. Data points are presented as mean values +/- SEM with an error ribbon, where n = 3.

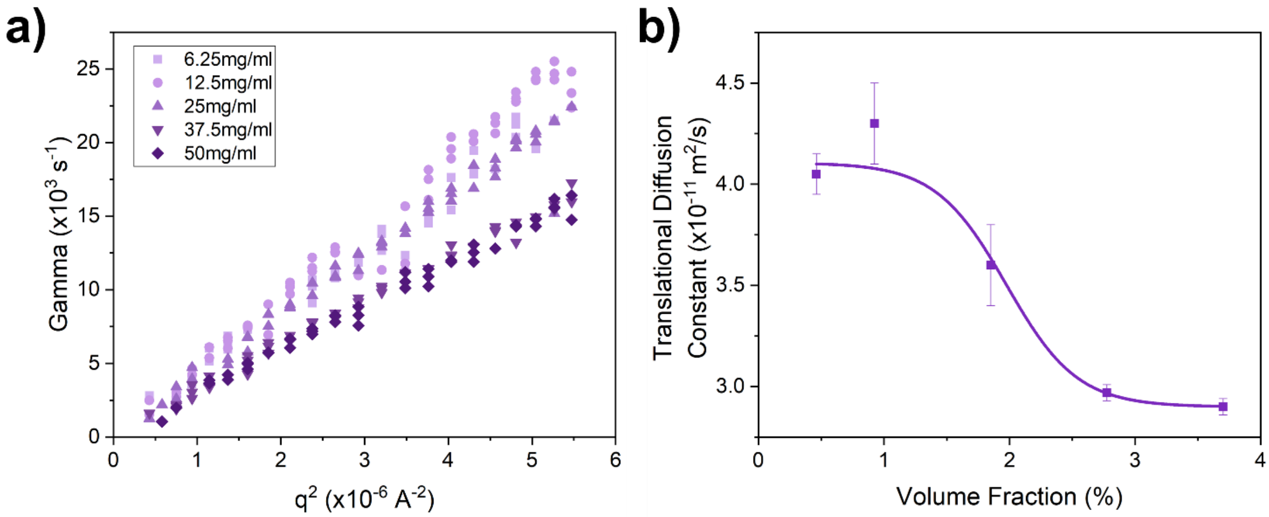

Supplementary Figure 15: a) The average decay rate, Gamma, extracted from DLS correlation data of pL_7 suspended at varying concentrations in buffer as a function of a q^2. **b)** The translational diffusion constant (the linear gradient of the data shown in panel a) as a function of pL_7 volume fraction. The solid line shows a sigmoidal fit to the data demonstrating a transition from the dilute to the semi-dilute regime. This transition is centred at (2 ± 0.2) % which is within error of the critical volume fraction extract from our rheology data (Fig. 3b, (1.5 ± 0.3) %) and in close agreement with the value predicted by our geometric model (Eqn 1, 1.6%). Data points are presented as the extracted gradient values from supplementary figure 14a, where the error bars are the error in the fitted gradient.

Supplementary Methods

Circular Dichroism Spectroscopy (CD)

Far-UV circular dichroism spectra of protein L monomer and polyproteins were acquired on a Chirascan plus circular dichroism spectrometer (Applied PhotoPhysics) with a bandwidth of 2 nm, a step size of 1 nm, and a commercially available cuvette (Hellma) with a path length of 0.1mm. The samples were held at 20°C during the measurement. The raw circular dichroism signal was converted to mean residue ellipicity using the equation below,

$$MRE\ (\deg cm^2 dmol^{-1}) = \frac{100 \cdot \theta\ (deg)}{(N-1) \cdot c \cdot l}$$

where θ, N, c and l are the degree of ellipticity, number of amino acids in the protein, molar concentration and the path length respectively.

Small-Angle X-ray Scattering (SAXS)

SAXS measurements were performed on an Anton Paar SAXSpace system (q-range of 0.01–0.7 $Å^{-1}$). pL construct samples were loaded into 1 mm path length borosilicate glass capillary tubes. Scattering curves were collected at 20°C. In order to extract the power law of the mid q-region, we fit the SAXS data with Beaucage's unified exponential/power law fit[5–7] (of the second order) which allows us to capture both the scattering profile of finite width of the polyprotein and the power law of the elongated dimension. Using this model independent fit, we extract the power law of the mid-q region which can give an insight into the shape of the scattering object.

Thermogravimetric analysis (TGA)

TGA measurements were performed on pL hydrogels constructed from varying AR building blocks at 37.5mg·ml^{-1} on TGA-50 Thermogravimetric Analyzer (Shimadzu Scientific Instruments). 40 µl of sample was gelated and loaded into a 70 µl aluminium crucible before heating, 50ml/min of nitrogen gas was flowed through the sample environment during the measurement. The samples were equilibrated at 20 °C before

heating to 200 °C at a rate of 5 °C/min and finally held isothermally at 200 °C for 2 mins. The percentage weight loss was calculated using the equation below,

$$\%\, Weight\ loss = \frac{Final\ Weight}{Initial\ Weight}\ x\ 100\%$$

Dynamic Light Scattering (DLS)

DLS measurements of pL7 at varying protein concentrations were conducted using a 3D Photo-correlation Spectrometer (LS Instruments) with a laser excitation wavelength of 660 nm. The samples were placed into 5mm diameter glass tubes and scattered light was detected at scattering angles, θ, between 30-135o in intervals of 5o, at a temperature of 25°C. The intensity autocorrelation functions, g2, were calculated from the time dependent scattered intensities and then subsequently converted to field autocorrelation functions, g1, using the Siegert relation:

$$g_1 = \sqrt{\frac{|g_2 - 1|}{\sigma}}$$

Here, σ is the set-up dependent coherence factor, ~0.95 for our spectrometer. The g1 data show two contributions: we attribute the faster process to the scattering from individual proteins and the slower to the scattering from aggregates. Thus, the data were fit using a sum of two stretched exponentials of the form:

$$g_1 = a_1 e^{\left(\frac{-t}{\tau_1}\right)^{\beta_1}} + a_2 e^{\left(\frac{-t}{\tau_2}\right)^{\beta_2}} + b\ ,$$

where a_i are the amplitudes, β_i are the stretching parameters, and τ_i are the characteristic timescales of the two contributions. The b parameter is the baseline amplitude.

The relaxation rate, Γ=1/τ is related to the self-diffusion coefficient, $\Gamma=Dq^2$, where q = 4πn/λ sin(θ/2), λ is the laser excitation wavelength and θ is the scattering angle. The D parameter for different concentrations of the pL polyprotein 7-mer could be determined through linear fits of the Γ vs q^2 data for the faster process observed in the autocorrelation functions. pL_7 at varying protein concentrations were conducted on a LS Spectrometer Variable Multi-Angle Light Scattering (LS Instruments). The samples were measured over multiple angles between 30-135° in intervals of 5°, and help at 20°C.

Code Repositories

BioNet simulations (Fig. 1c) were performed using BioNet, a software package in development at the University of Leeds. Kinetic lattice simulations were performed using a bespoke software also developed at the university of Leeds. Access to the code repositories can be found at https://doi.org/10.5518/1344.

pL polyprotein sequences

Below are the residue sequences for each of the pL polyprotein sequences.

Hexa-Histag (for purification)

TEV cleavage site (not used in this study)

Protein L Folded Domain

Linker Regions (unstructured protein L amino acids)

pL_x	Amino Acid Sequence	Molecular Mass, Mr (Daltons)	Extinction Coefficient, ε_{280} (M^{-1} cm^{-1})
pL	MHHHHHHGENLYFQGAMEEVTIKANLYFANGSTQTAEFKGTFEKATSE AYAYADTLKKDNGEWTVDVADKGYTLNIKFAG-	8998.89	12950
pL_2	MGSSHHHHHHGENLYFQGAMEEVTIKANLYFANGSTQTAEFKGTFEKA TSEAYAYADTLKKDNGEWTVDVADKGYTLNIKFAGAMEEVTIKANLYFA NGSTQTAEFKGTFEKATSEAYAYADTLKKDNGEWTVDVADKGYTLNIKF AG-	16116.76	24410
pL_3	MGSSHHHHHHGENLYFQGAMEEVTIKANLYFANGSTQTAEFKGTFEKA TSEAYAYADTLKKDNGEWTVDVADKGYTLNIKFAGAMEEVTIKANLYFA NGSTQTAEFKGTFEKATSEAYAYADTLKKDNGEWTVDVADKGYTLNIKF AGAMEEVTIKANLYFANGSTQTAEFKGTFEKATSEAYAYADTLKKDNGE WTVDVADKGYTLNIKFAG-	23465.83	35870
pL_4	MGSSHHHHHHGENLYFQGAMEEVTIKANLYFANGSTQTAEFKGTFEKA TSEAYAYADTLKKDNGEWTVDVADKGYTLNIKFAGAMEEVTIKANLYFA NGSTQTAEFKGTFEKATSEAYAYADTLKKDNGEWTVDVADKGYTLNIKF AGAMEEVTIKANLYFANGSTQTAEFKGTFEKATSEAYAYADTLKKDNGE WTVDVADKGYTLNIKFAGAMEEVTIKANLYFANGSTQTAEFKGTFEKAT SEAYAYADTLKKDNGEWTVDVADKGYTLNIKFAG-	30583.7	47330
pL_5	MGSSHHHHHHGENLYFQGAMEEVTIKANLYFANGSTQTAEFKGTFEKA TSEAYAYADTLKKDNGEWTVDVADKGYTLNIKFAGAMEEVTIKANLYFA NGSTQTAEFKGTFEKATSEAYAYADTLKKDNGEWTVDVADKGYTLNIKF AGAMEEVTIKANLYFANGSTQTAEFKGTFEKATSEAYAYADTLKKDNGE WTVDVADKGYTLNIKFAGAMEEVTIKANLYFANGSTQTAEFKGTFEKAT SEAYAYADTLKKDNGEWTVDVADKGYTLNIKFAGAMEEVTIKANLYFAN GSTQTAEFKGTFEKATSEAYAYADTLKKDNGEWTVDVADKGYTLNIKFA G-	37701.57	58700
pL_6	MGSSHHHHHHGENLYFQGAMEEVTIKANLYFANGSTQTAEFKGTFEKA TSEAYAYADTLKKDNGEWTVDVADKGYTLNIKFAGAMEEVTIKANLYFA NGSTQTAEFKGTFEKATSEAYAYADTLKKDNGEWTVDVADKGYTLNIKF AGAMEEVTIKANLYFANGSTQTAEFKGTFEKATSEAYAYADTLKKDNGE WTVDVADKGYTLNIKFAGAMEEVTIKANLYFANGSTQTAEFKGTFEKAT SEAYAYADTLKKDNGEWTVDVADKGYTLNIKFAGAMEEVTIKANLYFAN GSTQTAEFKGTFEKATSEAYAYADTLKKDNGEWTVDVADKGYTLNIKFA	44819.43	70250

	GAMEEVTIKANLYFANGSTQTAEFKGTFEKATSEAYAYADTLKKDNGEWTVDVADKGYTLNIKFAG-		
pL$_7$	MGSSHHHHHHGENLYFQGAMEEVTIKANLYFANGSTQTAEFKGTFEKATSEAYAYADTLKKDNGEWTVDVADKGYTLNIKFAGAMEEVTIKANLYFANGSTQTAEFKGTFEKATSEAYAYADTLKKDNGEWTVDVADKGYTLNIKFAGAMEEVTIKANLYFANGSTQTAEFKGTFEKATSEAYAYADTLKKDNGEWTVDVADKGYTLNIKFAGAMEEVTIKANLYFANGSTQTAEFKGTFEKATSEAYAYADTLKKDNGEWTVDVADKGYTLNIKFAGAMEEVTIKANLYFANGSTQTAEFKGTFEKATSEAYAYADTLKKDNGEWTVDVADKGYTLNIKFAGAMEEVTIKANLYFANGSTQTAEFKGTFEKATSEAYAYADTLKKDNGEWTVDVADKGYTLNIKFAGAMEEVTIKANLYFANGSTQTAEFKGTFEKATSEAYAYADTLKKDNGEWTVDVADKGYTLNIKFAG-	51937.30	81710

Supplementary References

1. Weisel, J. W. & Litvinov, R. I. *Fibrin Formation, Structure and Properties*. (Springer, Cham, 2017).
2. Yang, Z., Mochalkin, I. & Doolittle, R. F. A model of fibrin formation based on crystal structures of fibrinogen and fibrin fragments complexed with synthetic peptides. *Proc. Natl. Acad. Sci.* **97**, 14156–14161 (2000).
3. Litvinov, R. I. & Weisel, J. W. Fibrin mechanical properties and their structural origins. *Matrix Biol.* **60–61**, 110–123 (2017).
4. Lattuada, M., Wu, H., Hasmy, A. & Morbidelli, M. Estimation of Fractal Dimension in Colloidal Gels. *Langmuir* **19**, 6312–6316 (2003).
5. Beaucage, G. Approximations Leading to a Unified Exponential/Power-Law Approach to Small-Angle Scattering. *J. Appl. Crystallogr.* **28**, 717–728 (1995).
6. Beaucage, G. Small-Angle Scattering from Polymeric Mass Fractals of Arbitrary Mass-Fractal Dimension. *J. Appl. Crystallogr.* **29**, 134–146 (1996).
7. Hammouda, B. Analysis of the Beaucage model. *J. Appl. Crystallogr.* **43**, 1474–1478 (2010).
